# Behavioral Selection of Coprophagy in an Arid‐Adapted Herbivore: Does a Compatibility–Risk Gradient Shape Selective Coprophagy?

**DOI:** 10.1002/ece3.73444

**Published:** 2026-04-07

**Authors:** Christin Moeller, Scott E. Henke, Sandra Rideout‐Hanzak, Cord B. Eversole

**Affiliations:** ^1^ Caesar Kleberg Wildlife Research Institute Texas A&M University‐Kingsville Kingsville Texas USA; ^2^ Arthur Temple College of Forestry and Agriculture Stephen F. Austin State University Nacogdoches Texas USA

**Keywords:** adaptive behavior, arid ecosystems, behavioral ecology, digestive efficiency, foraging trade‐offs, microbial homeostasis, nutrient cycling, symbiotic regulation

## Abstract

Coprophagy is widespread among herbivores but remains poorly understood in reptiles, where its ecological function has rarely been tested. We conducted controlled choice experiments with 25 adult Texas tortoises ( *Gopherus berlandieri* ), an arid‐adapted hindgut fermenter, to determine whether feces consumption represents incidental ingestion or a selective behavioral strategy. Each tortoise was presented with six feces types representing self, conspecific, and heterospecific sources (feral hog ( *Sus scrofa* ), raccoon ( *Procyon lotor* ), coyote ( *Canis latrans* ), and nilgai ( *Boselaphus tragocamelus* )). Coprophagy occurred in 96% of individuals, and both the probability and relative amount of consumption differed significantly among feces types. Tortoises showed a consistent preference hierarchy (e.g., self, conspecific, feral hog, raccoon, coyote, nilgai), providing evidence for a compatibility–risk gradient in which individuals favored feces most similar in dietary composition and microbial origin while avoiding those likely to pose digestive or pathogenic risk. These patterns suggest that coprophagy serves as a behavioral mechanism of nutrient recapture and microbial maintenance, sustaining fermentation efficiency in environments where microbial reservoirs are ephemeral. By selectively regulating microbial exposure, tortoises mitigate ecological constraints imposed by aridity and low nutrient availability. Our findings identify selective coprophagy as an adaptive behavior that links individual physiology with nutrient and microbial cycling in arid ecosystems, illustrating how behavioral flexibility promotes persistence in long‐lived vertebrates inhabiting resource‐limited environments.

## Graphical abstract

Estimated amount of each scat type consumed by Texas tortoises ( *Gopherus berlandieri* ) during 48‐h feeding trials expressed as area under the cumulative‐consumption curve (AUC; ±95% CI). Coprophagy is prevalent in Texas tortoises, and tortoises selectively choose feces along a compatibility‐risk gradient, which is evolutionarily meaningful.

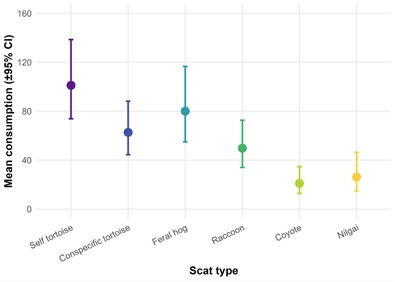

## 1 Introduction

In arid and nutrient‐poor environments, herbivores face chronic energetic and nutritional constraints (Noy‐Meir 1973; Owen‐Smith 1988; Illius and O'Connor 1999; Pellegrini 2016). Seasonal drought, low primary productivity, and the poor digestibility of fibrous forage limit nutrient intake and microbial stability within the gut (Abraham et al. 2019; Bondaruk et al. 2022). To persist under these conditions, herbivores often employ behavioral and physiological strategies that conserve or recapture scarce nutrients, maintain digestive function, and minimize energetic waste (Foley et al. 1998; White 2011). Such strategies include re‐ingestion of food residues, selective feeding on microbially enriched substrates, and utilization of mineral‐rich soil or bone (Hirakawa 2001). These behaviors characterize behavioral nutrient recapture, which is the active recovery of nutritional or symbiotic resources that would otherwise be lost from the organism's internal nutrient cycle (Sterner and Elser 2002; Raubenheimer et al. 2009). Besides nutrient recapture, fecal ingestion of gastroliths that contain soil, stones, shells, and bones, may be consumed for endoparasite removal, stomach cleaning, and mineral supplementation (e.g., calcium; Moore and Dornberg 2014). Reptiles that eat plant‐based diets are commonly mineral‐deficit so need to supplement their diets with minerals (Wallach 1971).

One of the most prominent examples of behavioral nutrient recapture is coprophagy (i.e., the deliberate consumption of feces). Coprophagy is widespread among vertebrates, particularly in herbivores and omnivores reliant on hindgut fermentation, where critical nutrients and microbial products are produced beyond the site of absorption (Hirakawa 2001; Stevens and Hume 2004). In lagomorphs and rodents, coprophagy enhances nitrogen and vitamin recovery through re‐ingestion of microbially enriched cecotropes (Hirakawa 2001; Sakaguchi 2003). Similarly, some ungulates and primates consume conspecific or heterospecific feces to supplement minerals, microbial symbionts, or residual plant nutrients (Spitzer et al. 2023; Masi and Breuer 2018). Although coprophagy is often interpreted as a response to nutritional deficiency (Hirakawa 2001), it can also function as a routine behavioral mechanism for microbial maintenance and digestive homeostasis, especially in species feeding on high‐fiber, low‐nitrogen diets (Hirakawa 2001; Stevens and Hume 2004).

In reptiles, coprophagy has been noted in a variety of taxa (Bjorndal 1997), but remains poorly studied in functional or ecological contexts. Tortoises (i.e., Testudinidae), as slow‐metabolism, hindgut‐fermenting herbivores, are particularly likely to benefit from re‐ingestion of feces. By reintroducing microbial communities and residual nutrients, coprophagy may increase digestive efficiency and stabilize gut symbioses that enable digestion of cellulose (Bjorndal 1997). In hatchling tortoises, ingestion of adult feces facilitates initial colonization of beneficial gut flora (Troyer 1982; Yuan et al. 2015), and similar behavior in adults could serve to maintain these microbial assemblages through seasonal dietary shifts or dehydration stress. At the same time, fecal consumption may carry significant risks, such as parasites and pathogens, especially when consuming feces from carnivores (Kazacos 2001). Thus, coprophagy likely involves a trade‐off between nutritional or probiotic gain and pathogen exposure, with selective discrimination among fecal sources minimizing risk while maximizing benefit. Therefore, a compatibility‐risk gradient may exist, where tortoises select the most compatible fecal material to ingest and acquire a probiotic gain with the least negative risk of exposure to pathogens and parasites.

Despite its theoretical and ecological relevance, coprophagy in reptiles, particularly in tortoises, remains largely anecdotal. Mentions of feces consumption in tortoises appear in dietary observations (Scalise 2011; Rose and Judd 2014) but lack quantitative evidence or analysis of selectivity. We acknowledge that a knowledge gap exists concerning the coprophagous behavior of tortoises and the reasons for this behavior. This gap is surprising given that tortoises inhabit nutrient‐scarce habitats and exhibit digestive physiology strongly reliant on microbial fermentation (Bjorndal 1997). However, microbial maintenance to aid digestion is a hypothesis to explain this behavior rather than an established function in reptiles. Understanding the prevalence and selectivity of coprophagy in tortoises can provide insight into the behavioral mechanisms by which long‐lived herbivores maintain nutrient balance and gut symbiosis in extreme environments.

The Texas tortoise ( *Gopherus berlandieri* ), an arid‐adapted herbivore endemic to south Texas and northeastern Mexico, offers a model for examining the mechanism of behavioral nutrient recapture via coprophagy. Populations of Texas tortoises occur in semi‐arid thornscrub and grassland ecosystems characterized by seasonal drought, poor soil fertility, and variable plant productivity (Rose and Judd 1975; Kazmaier et al. 2001). The species feeds primarily on grasses, forbs, and succulents such as prickly pear (*Opuntia* spp.), but also consumes mineralized substrates and animal matter (i.e., mammal hair, insects, snail shells, feathers; Scalise 2011; Rose and Judd 2014). Given its reliance on hindgut fermentation and its occurrence in nutrient‐limited habitats, Texas tortoises likely exhibit behaviors that facilitate nutrient recapture and digestive stability.

Therefore, we used this species as a representative model to experimentally test the ecological function of coprophagy in an arid environment. Specifically, we conducted a controlled cafeteria‐style experiment to quantify both the prevalence and selectivity of coprophagy among six scat types differing in trophic origin and potential nutritional or pathogenic properties. Our experiment addressed three primary questions: (1) How common is coprophagy among individuals under controlled conditions? (2) Does the probability and amount of feces consumed differ among scat types, including self, conspecific, and heterospecific sources? (3) Are preference hierarchies consistent across consumption likelihood, quantity eaten, and first‐choice behavior?

We hypothesized that coprophagy functions as an adaptive strategy of nutrient recapture in arid‐adapted herbivores, balancing the recovery of nutritional resources with avoidance of feces carrying higher pathogen risk. Accordingly, we predicted that tortoises would preferentially consume feces most similar to their own diet (e.g., self, conspecific, and herbivore feces) while discriminating against feces of omnivores and carnivores that offer lower nutritional return or greater disease risks. Through this experiment, we aimed to demonstrate and explain the adaptive role of coprophagy as an ecological and physiological strategy for nutrient conservation in arid environments.

## 2 Methods

### 2.1 Study Area and Specimen Collection

We used the Texas tortoise as a representative arid‐adapted herbivore to test behavioral nutrient recapture through selective coprophagy. We collected tortoises for a translocation study from a Liquid Natural Gas (LNG) property near Port Isabel, southern coastal Texas, USA, between June and October 2022 via road cruising, systematic searches, the aid of a detector dog, and fortuitous encounters (Moeller, Elissetche, et al. 2025). Captured tortoises were marked with a unique individualized identification number that corresponded to their capture order, which entailed filing a triangular notch in its marginal scutes according to the procedures of Cagle (1939). Tortoises were then sexed using sexual dimorphic characteristics of plastron concavity, gular length, and anal scute width (McRae et al. 1981; Eubanks et al. 2003), and weighed to the nearest gram with a spring‐loaded scale. Carapace and plastron length were measured to the nearest millimeter (mm), as well as maximum width and height using calipers, and circumference using a tailor's tape. Age of tortoises was estimated based on carapace length as established by Hellgren et al. (2000) and Kazmaier et al. (2002), which delineated varying growth rates across different age cohorts. Health status was considered optimal if tortoises displayed no clinical symptoms of upper respiratory tract disease (URTD), and demonstrated vitality and strength in daily movements.

### 2.2 Experimental Design

After each collection trip to the LNG property, collected tortoises were transported by vehicle and maintained in 1.2 × 1.8 × 2.0 m individual cages at an isolation holding facility at the Caesar Kleberg Wildlife Research Institute Duane M. Leach Aviary facility to assess tortoise health for URTD (Moeller, Perales, et al. 2025). The walls of each pen were lined with 22 mils vinyl‐coated polyester tarp to eliminate direct contact between tortoises and spread of possible pathogens. Alfalfa hay was used as bedding and also could be eaten by tortoises. Tortoises were provided Mazuri tortoise low‐starch, pelleted diet (Mazuri Exotic Animal Nutrition, St. Louis, Missouri 63,166) and water *ad libitum*. Tortoise water intake was subsidized by providing diced fresh cucumber (*Cucumis sativis*) and watermelon ( *Citrullus lanatus* ) fruit, and tortoises were provided shallow 100 × 100 × 2 cm soaking trays filled with water. Each pen was equipped with a 3‐sided 40 × 40 × 20 cm escape box with a wooden top for added shelter, a Fluker (Fluker Farms, Port Allen, Louisiana 70,767) 150‐W ceramic heat emitter bulb, and a Reptisun (Zoo Med Laboratories Inc., San Luis Obispo, California 93,401) UVA UVB Reptile 23 W fluorescent lamp. The heat lamps and UV lights were provided *ad libitum* and tortoises were free to move underneath or away from both devices as needed. Twenty‐five healthy adult tortoises (12 M : 13F) were randomly selected from the 171 tortoises collected from the LNG property (Moeller, Elissetche, et al. 2025) to be subjects in this study. Tortoise selection criteria were adult‐sized tortoise (i.e., carapace range of 160–180 mm), healthy appearance (i.e., no physical injuries, no runny eyes or nares consistent with signs of mycoplasma infection; Moeller, Perales, et al. 2025), and even sex ratio.

We collected scats daily from tortoises held in individual cages and placed scats in marked bags to identify the tortoise from which the scats came. Tortoise cages were checked every 8 h for scat to maintain scat as fresh as possible. Cages were cleaned each morning and food refreshed to provide tortoises with a consistent maintenance schedule. We also collected fresh scat from trapper‐collected raccoons ( *Procyon lotor* ), coyotes ( *Canis latrans* ), and feral hogs ( *Sus scrofa* ). We accompanied the trapper during his morning trap checks and performed necropsies on dispatched animals. Fresh feces were removed from the large intestines and placed in paper bags marked for species. Lastly, we collected fresh nilgai ( *Boselaphus tragocamelus* ) scat from nilgai on the LNG property. We followed nilgai until we saw them defecate. We immediately collected the fresh scat and placed the scat in marked paper bags. All scat was immediately placed in a portable freezer (−20°C), transported to an ultracold (−80°C) freezer, until the beginning of the experiment, at which point, scats were thawed. Therefore, all scat types were collected immediately (or within 8 h of defecation in the case of Texas tortoises) to reduce desiccation of feces, and immediately frozen to maintain freshness, flavor, and nutritional value (https://www.fsis.usda.gov/food‐safety/safe‐food‐handling‐and‐preparation/food‐safety‐basics/freezing‐and‐food‐safety). Freezing does not generally kill most microbes, but instead, causes bacteria, yeast, and molds to enter into a dormant or hibernation state, from which the microbes become active again upon thawing (https://www.daymarksafety.com). Scat type was selected based upon compatibility of fecal composition to tortoises (i.e., plant‐based diet [e.g., Texas tortoises, nilgai, feral hogs, and to a lesser‐extent raccoons and coyotes]) and scat abundance within tortoise habitat. Many tortoise species and predators (i.e., raccoons and coyotes) utilize roads and animal paths as movement corridors (Frey and Conover 2006; Hromada et al. 2020) and often defecate along such corridors. Raccoons and nilgai develop latrines for defecation (Page et al. 1999; Zoromski et al. 2022); therefore, the quantity of potential scat within the environment increased the likelihood of tortoise encounter. Although other reptile species, such as Texas horned lizards ( *Phrynosoma cornutum* ) and Indigo snakes ( *Drymarchon couperi* ) were abundant within the collection area, their fecal composition (i.e., ants and carnivorous diet, respectively) were considered less compatible to Texas tortoises. White‐tailed deer ( *Odocoileus virginianus* ) and bobcats ( *Lynx rufus* ) occurred within the collection area, but their feces were not commonly encountered during tortoise collections. Therefore, we selected the most composition‐compatible and abundant feces to test coprophagy behavior in Texas tortoises.

At the onset of the experiment, alfalfa hay was removed from cages so we could measure tortoise consumption of foods and scats. We developed a cafeteria‐style experiment with 20 g of each feces type placed in individual shallow non‐tip and non‐skid 236‐mL stainless steel metal bowls (Outdoor Dog Supply, Chesapeake, Virginia 23,322). Only one feces type was placed in individual metal bowls, and bowls were marked for feces type to aid in scat identification. We did not analyze each feces type for nutritional content, which we acknowledge could have affected fecal consumption. Instead, we provided an equal amount by mass of each feces type in an attempt to reduce selection bias based on quantity. In addition, 100 g of Mazuri tortoise low‐starch, pelleted diet and 100 g of an equal mix of diced cucumber and watermelon were placed in separate metal bowls. All bowls were placed in a straight line and spaced 10 cm apart in random order at the back of the cage. Coprophagy observations were recorded using Geeni Vivid Indoor Smart Wi‐Fi Security Cameras (Merkury Innovations, New York, NY) positioned centrally above each feeding station. Cameras provided continuous video footage of tortoise activity, and recordings were archived and reviewed post‐trial for data analysis. At 12 h intervals up to 48 h, we reweighed the quantity of food and scat in each bowl and re‐randomized the order of bowl placement. Bowls were re‐randomized each 12‐h period to determine if tortoises were selecting for bowl position rather than bowl contents. Because the tortoises were maintained in a pavilion‐style building that was exposed to outdoor humidity conditions, we exposed 20 g of each feces type and 100 g of food types to the environment in an animal‐proof cage to determine weight change as a result of water gain or loss in feces and food, and accounted for this change within consumption rates. For example, if 20 g of raccoon feces only weighed 19.35 g (i.e., 96.75% of original weight) during the next 12‐h period due to desiccation, then consumption of raccoon feces was based upon 96.75% of the previous weight.

We plotted cumulative consumption of each scat type for each tortoise with scat consumption (in grams) on the y‐axis and time (hrs; 12, 24, 36, and 48 h) on the x‐axis. We calculated the area under the cumulative consumption curve using the Rodgers' index for cafeteria style experiments (Krebs 1989), which calculates each scat consumption through time as a single measurement. For example, if the entire scat type (20‐g) was consumed within the first 12‐h time interval, the area under the curve would be the maximum or 840 g‐hour units, which we standardized to 1.0. In this case, all 20 g of feces were consumed during the first 12‐h interval. Therefore, the area under the cumulative consumption curve is calculated as: [0.5 (20 g × 12 h) + (20 g × 12 h) + (20 g × 12 h) + (20 g × 12 h)] = 840 g‐h, which was standardized to be 1.0. The latter three (20 g × 12 h) represents the 24‐h, 36‐h, and 48‐h cumulative consumption periods. By way of a second example, if no feces was consumed by a tortoise during the first 12‐h period, but 10 g, 2 g, and 4 g were consumed during the 24‐h, 36‐h, and 48‐h periods, respectively, then the cumulative consumption area would be calculated as the sum of: 0, 0.5 (10 g × 12‐h), [0.5 (2 g × 12‐h) + (10 g × 12‐h)], and [0.5 (4 g × 12‐h) + (12 g × 12‐h)] for the 12‐h, 24‐h, 36‐h, and 48‐h time intervals, respectively, or 360 g‐hrs. The cumulative area would then be standardized using 840 g‐h as the maximum, or 360/840 g‐h = 0.43. For analysis and reporting, the area under the cumulative consumption curve (AUC) was expressed in gram‐hour (g·h) units, representing the integrated mass of feces consumed through time. The theoretical maximum was 840 g·h (complete consumption within 12 h). Although these values are directly comparable across treatments, they are not grams of feces consumed but a continuous preference index quantifying both timing and amount of intake (Rodgers 1984).

### 2.3 Data Analysis

Because each tortoise was exposed to all six scat types, our experiment followed a repeated‐measures design rather than a set of independent replicates. This is not pseudoreplication in the classical sense (Hurlbert 1984), but it does require accounting for within‐individual non‐independence. Individual tortoises represent the experimental units, as treatments were applied and responses measured repeatedly within the same individuals. Each tortoise contributed one preference value (Rodgers' index) per scat type, resulting in correlated observations within individuals. Response variables included area under the consumption curve (AUC; g·h) per scat type and individual tortoise as a measure of relative amount consumed, and binary consumption outcomes (consumed = 1, not consumed = 0) for analyses of consumption probability.

We analyzed AUC values using a generalized linear mixed‐effects model (GLMM) with a Gamma error distribution and log link, appropriate for continuous, positive, and right‐skewed response data (McCullagh and Nelder 1989; Bolker et al. 2009). Binary consumption outcomes were analyzed using a binomial GLMM with a logit link, appropriate for modeling probabilities of discrete events (McCullagh and Nelder 1989; Bolker et al. 2009). In both models, scat type and sex were included as fixed effects, and individual tortoise identity was included as a random intercept to account for repeated measures and within‐individual non‐independence. Scat type represents the within‐subject (repeated) factor, while tortoise identity represents the experimental unit and the source of random variation among individuals.

Models were fit in R (version 4.5.1; R Core Team 2025) using the lme4 package (Bates et al. 2015). Model fit and assumptions were evaluated using residual diagnostics, including assessments of residual distributions, checks for overdispersion, and identification of outliers or other deviations from model assumptions (Bolker et al. 2009). No substantial violations of model assumptions were detected. Significance of fixed effects was assessed using *F*‐tests via the lmerTest package (Kuznetsova et al. 2017). Where significant main effects or interactions occurred, we conducted pairwise comparisons with Tukey's HSD adjustment using the emmeans package (Lenth 2023). All results were considered significant at *α* = 0.05.

To test whether tortoises were more likely than expected by chance to consume particular scat types first, we performed a chi‐square goodness‐of‐fit test comparing observed frequencies of first choices to those expected under a random (equal‐frequency) distribution. All means are reported ±1 SE using non‐ranked values for interpretability.

## 3 Results

Of the 25 tortoises tested, 24 (96%) consumed 2–4 scat types during the 48‐h experiment. On average, individuals consumed 3.2 ± 0.2 scat types (range = 2–4), with consumption frequencies highest for self, other tortoise, and feral hog feces (Table 1). No tortoise ate only 1 scat type, nor did a tortoise eat 5 or all 6 scat types during the 48‐h experiment. Overall, 20 (80%), 17 (68%), 14 (56%), 14, 8 (32%), and 6 (24%) of the 25 tortoises consumed some of their own scat, conspecific scat, and raccoon, feral hog, coyote, and nilgai scat, respectively.

**TABLE 1:** Model‐estimated probability and amount of consumption of six scat types by Texas tortoises ( *Gopherus berlandieri* ) during 48‐h feeding trials. Values are estimated marginal means ± standard error (SE) and 95% confidence intervals (CI) from generalized linear mixed models: A binomial GLMM for the probability of consumption and a Gamma GLMM for the amount consumed (amount consumed (AUC = area under the cumulative consumption curve (Rodgers' index) expressed in gram‐hour (g·h) units; maximum = 840 g·h = complete consumption within 12 h)). Letters indicate Tukey‐adjusted groups that did not differ significantly (*p* > 0.05).

Scat type	Probability (±SE)	Group	95% CI (probability)	Mean area under the curve (AUC; g·h ± SE)	95% CI (amount)	Group
Self‐tortoise ( *G. berlandieri* )	0.80 ± 0.08	A	0.66–0.89	101.2 ± 16.2	73.9–138.6	A
Conspecific tortoise ( *G. berlandieri* )	0.68 ± 0.09	AB	0.49–0.86	62.7 ± 10.9	44.5–88.2	A C
Feral hog ( *Sus scrofa* )	0.56 ± 0.10	ABC	0.37–0.74	80.1 ± 15.4	55.0–116.6	A
Raccoon ( *Procyon lotor* )	0.56 ± 0.10	ABC	0.37–0.74	49.8 ± 9.6	34.1–72.7	AB
Coyote ( *Canis latrans* )	0.32 ± 0.09	BC	0.18–0.51	21.1 ± 5.4	12.9–34.7	B
Nilgai ( *Boselaphus tragocamelus* )	0.24 ± 0.09	C	0.12–0.45	26.2 ± 7.7	14.7–46.5	BC

### 3.1 Probability of Consumption

**FIGURE 1:**
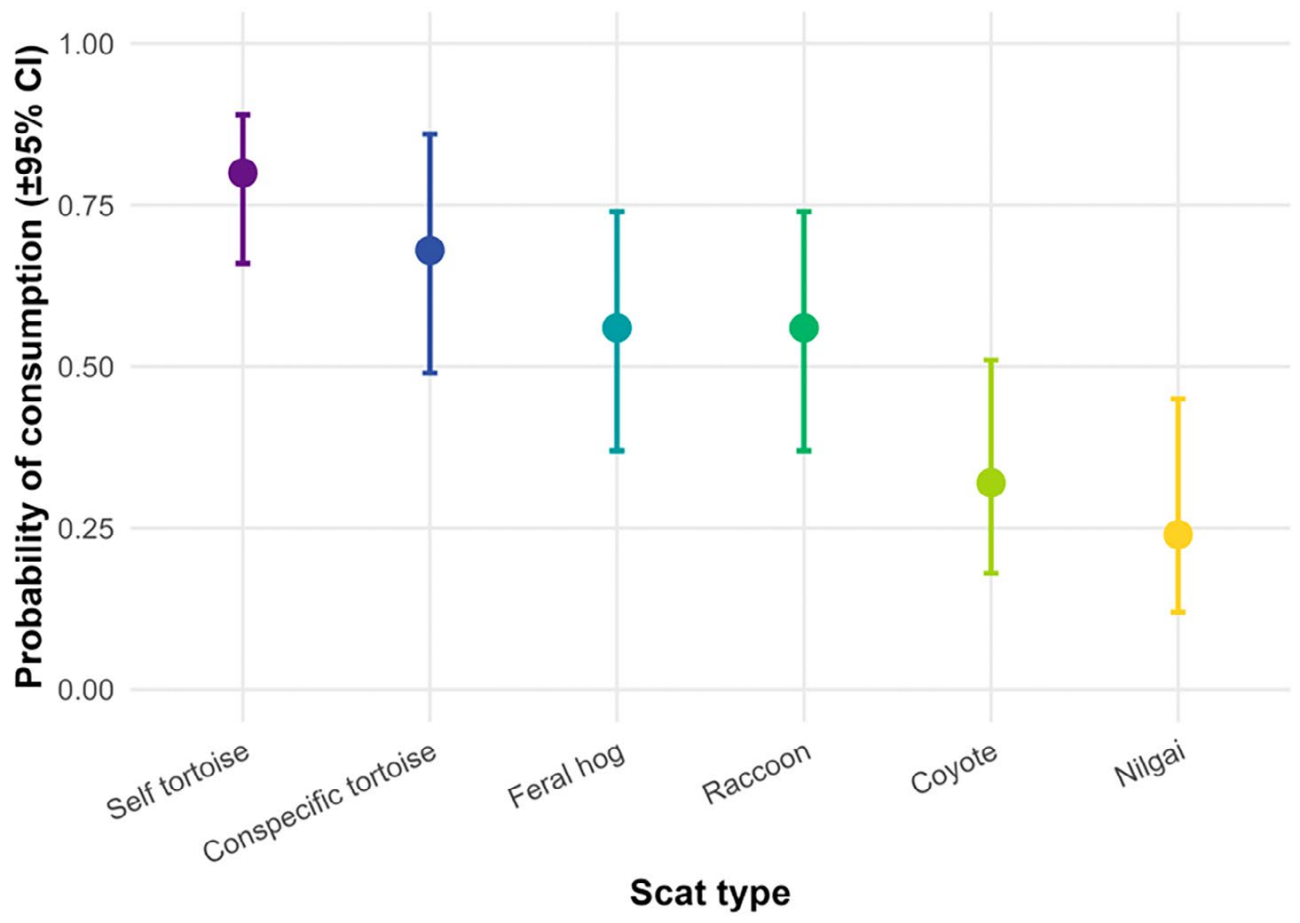
Probability of consumption of six scat types by Texas tortoises ( *Gopherus berlandieri* ) during 48‐h feeding trials. Plotted values are model‐based estimates (with confidence intervals) from the binomial and Gamma GLMMs (R Core Team 2025).

The probability that a given scat type was consumed varied significantly among treatments (*χ* ^2^ = 20.32, df = 5, *p* = 0.001; Figure 1). Neither sex (*χ* ^2^ = 0.15, df = 1, *p* = 0.70) nor the scat type × sex interaction (*χ* ^2^ = 0.95, df = 5, *p* = 0.97) affected the likelihood of feeding. Estimated marginal means indicated that tortoises were most likely to consume their own feces (probability = 0.80 ± 0.08 SE), followed by feces of other tortoises (0.68 ± 0.09), feral hogs (0.56 ± 0.10), raccoons (0.56 ± 0.10), coyotes (0.32 ± 0.09), and nilgai (0.24 ± 0.09). Post hoc comparisons (Tukey‐adjusted) showed that self and conspecific feces were consumed significantly more often than coyote (*p* = 0.015) or nilgai feces (*p* = 0.003), whereas consumption of feral hog and raccoon feces was intermediate and did not differ significantly from either the high‐preference (self and conspecific) or low‐preference (coyote and nilgai) groups (*p* > 0.20 for all comparisons; Table 1).

### 3.2 Relative Amount Consumed (AUC; g·Hr)

Texas tortoises consumed, on average, 108.2 ± 4.2 g (range = 54–149 g) of food and feces during the 48‐h trial, of which, on average, 98.8 ± 3.9 g (range = 48–135 g) was food while 9.4 ± 0.6 g (range = 0–14 g) comprised fecal matter. On average, fecal matter comprised 9.0% of the tortoise diet, of the 24/25 (96%) tortoises that practiced coprophagy.

**FIGURE 2:**
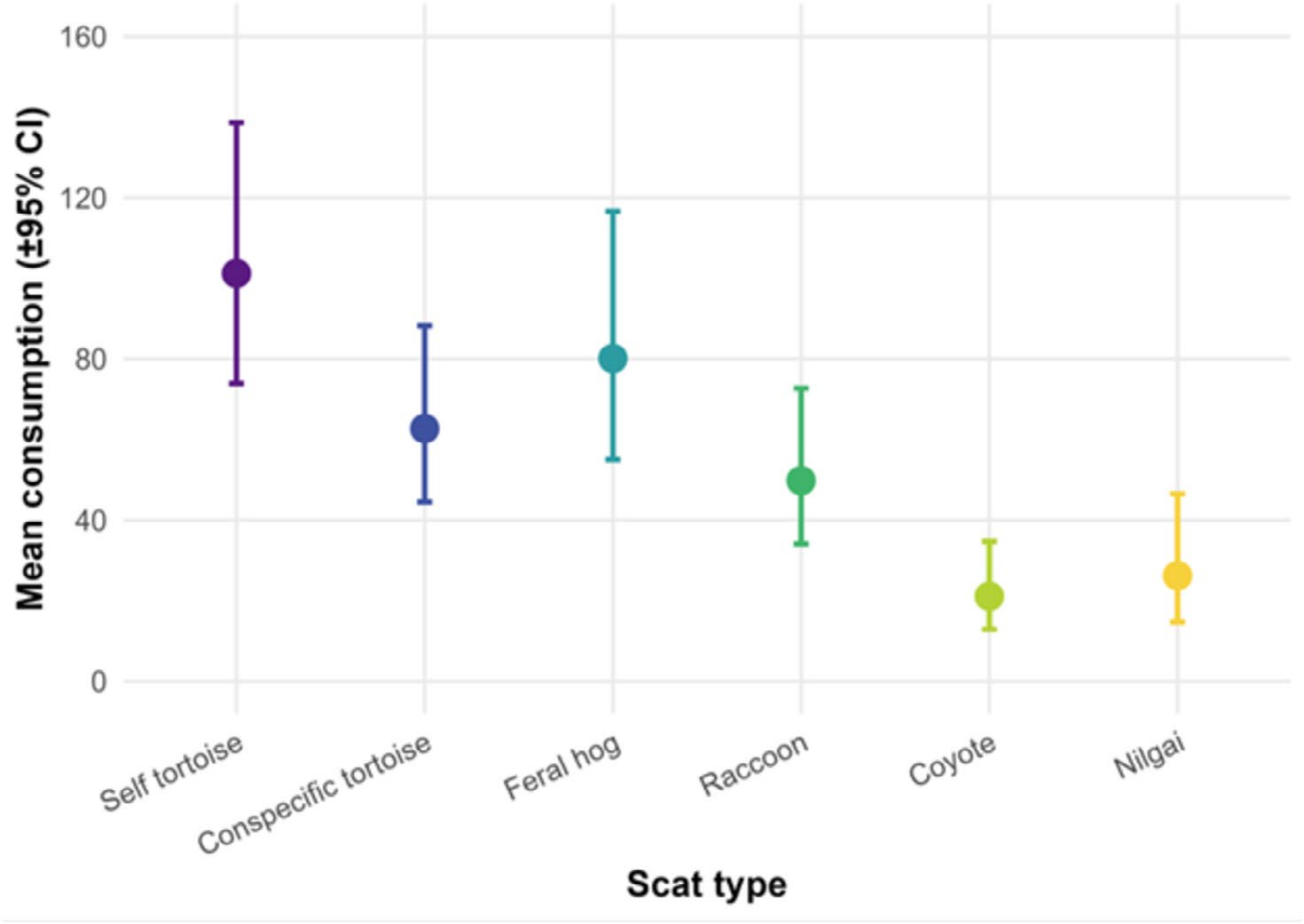
Amount of each scat type consumed by Texas tortoises ( *Gopherus berlandieri* ) during 48‐h feeding trials. Plotted values are model‐based estimates (with confidence intervals) from the binomial and Gamma GLMMs (R Core Team 2025).

Conditional on a scat type being eaten, the relative amount consumed (area under the cumulative consumption curve; preference index) also differed among scat types (*χ* ^2^ = 38.87, df = 5, *p* < 0.001; Table 1 and Figure 2). Sex (*χ* ^2^ = 0.58, df = 1, *p* = 0.44) and the scat type × sex interaction (*χ* ^2^ = 3.47, df = 5, *p* = 0.63) were not significant. Mean consumption (AUC ± SE; g·h) was highest for self‐tortoise feces (101.2 ± 16.2), followed by feral hog (80.1 ± 15.4), other tortoise (62.7 ± 10.9), raccoon (49.8 ± 9.6), nilgai (26.2 ± 7.7), and coyote feces (21.1 ± 5.4). Post hoc tests indicated that self and feral hog feces were consumed in greater quantities than coyote and nilgai feces (*p* < 0.001), with raccoon and other tortoise feces intermediate (*p* > 0.10; Table 1).

### 3.3 Consumption Sequence

Across treatments, both the likelihood of consumption and the amount consumed mapped onto a common ranking (e.g., self, conspecific, hog, raccoon, coyote, nilgai) indicating a consistent preference hierarchy across metrics. The order in which feces were first consumed differed from random expectation (*χ* ^2^ = 18.0, df = 5, *p* = 0.003). Half of all tortoises (50%) consumed their own feces first, and only a small proportion initiated feeding with coyote or nilgai scat (i.e., < 10%). No tortoise consistently fed from the same bowl position across observation intervals, indicating that choices reflected scat type rather than bowl placement.

## 4 Discussion

Coprophagy was nearly universal among Texas tortoises, with 96% of individuals consuming at least one fecal type and most sampling multiple sources. This high prevalence, combined with consistent selectivity among types, demonstrates that coprophagy in Texas tortoises is not incidental but a structured component of their foraging behavior. The ranked order of consumption (e.g., self, conspecific, feral hog, raccoon, coyote, nilgai) reveals a repeatable hierarchy reflecting nutritional and microbial compatibility as well as avoidance of physiological or pathogenic risk. These choices form a consistent decision framework that provides insight into how a long‐lived, slow‐metabolizing reptile manages nutrient limitation and microbial stability in arid environments. We do acknowledge that, to date, it is undocumented if microbes would survive passage through the digestive tract and successfully colonize within the adult gut of a tortoise. However, we propose the microbial gain through coprophagy as a hypothesis rather than a demonstrated function.

The ordered pattern of consumption indicates that feces selection follows a compatibility–risk gradient, favoring feces that maximize microbial and dietary similarity while minimizing pathogen exposure or digestive incompatibility. Feces from self and conspecifics, which most closely match the tortoise's gut microbiota and plant‐based diet, were consumed most readily and in greatest quantity. Feral hog and raccoon feces formed an intermediate tier of preference, reflecting partial dietary overlap (e.g., particularly succulents, fruits, and forbs), while coyote and nilgai feces, characterized by lower digestibility or reduced plant overlap with tortoise diets, or pathogen risk, were largely avoided. Many collected raccoons contained the fruit of prickly pear in their lower colon (i.e., identifiable by the purple color of the feces), which also is a common diet item of Texas tortoises (Scalise 2011). Although nilgai antelope consume a plant‐based diet, nilgai are ruminants that use a foregut process of digestion that ferment fiber before the stomach allowing for microbial protein absorption, while tortoises use a hind‐gut digestion process. The anaerobic fungi and bacterial communities found in tortoise microbiomes are distinct from those in ruminants (Pratt et al. 2023). It is possible that such a distinction caused tortoises to avoid nilgai feces (i.e., 6/25; 24% of tortoises consumed nilgai feces and of those tortoises, feces from nilgai was on average 12% of the fecal matter consumed by tortoises).

This consistency among individuals implies that tortoises employ reliable chemical and olfactory cues to evaluate fecal suitability, balancing potential nutritional or microbial gain against disease risk, a trade‐off also observed across other vertebrates (Hirakawa 2001; Weinstein et al. 2018; Videvall et al. 2023). For example, ungulates risk parasite exposure at mineral licks (Plummer et al. 2018; Severud et al. 2023; Utaaker et al. 2023), birds risk increased exposure to pollutants, pathogenic microbes, and antibiotic‐resistant microbes that can reduce host health for gut microbiome gains to obtain nutritional and energetic requirements (Dunbar et al. 2024), and primates accept insect‐borne pathogens for nutritional reward from fruits (Sarabian et al. 2018; dos Santos‐Barnett et al. 2022). Texas tortoises thus exemplify a broader behavioral principle (i.e., an extension of optimal foraging theory; MacArthur and Pianka 1966; Emlen 1966; Charnov 1976; Pyke et al. 1977; Stephens and Krebs 1986) of a vertebrate species integrating multiple cues to optimize benefit while minimizing cost under chronic ecological constraint.

From an evolutionary perspective, such selective feces ingestion may represent a behavioral solution to one of the central problems of survival in nutrient‐poor environments (e.g., maintaining symbiotic function without compromising immune defense). In arid systems where beneficial microbial exposure is both essential and risky, selection likely favored decision rules that optimize this trade‐off (Coleine et al. 2024; Wang et al. 2024; Hart 2011). The observed hierarchy, structured by dietary and microbial similarity, therefore likely reflects an evolved response to persistent environmental pressure, rather than opportunity (Lim and Bordenstein 2020; Youngblut et al. 2019; Rojas et al. 2021). Preference for self and conspecific feces supports the conclusion that coprophagy serves to maintain gut microbial continuity (Troyer 1982; Kohl et al. 2014; Videvall et al. 2023). For hindgut‐fermenting reptiles, digestive efficiency depends on the stability of cellulolytic and fermentative microbes (Troyer 1982; Bjorndal 1997; Karasov 2013). Autocoprophagy likely re‐inoculates the gut following defecation or feeding dormancy (Hirakawa 2001; Stevens and Hume 2004; Kohl et al. 2014), while allocoprophagy enables microbial exchange among individuals exploiting similar vegetation (Troyer 1982; Schwarm et al. 2009; Videvall et al. 2023). These behaviors suggest a self‐reinforcing feedback loop of microbial renewal that buffers digestion against seasonal shifts in plant composition or moisture availability. In this way, tortoises actively regulate their own microbial ecology through behavior, in which exposure is managed selectively rather than avoided entirely.

Behaviorally sustaining microbial diversity may be particularly advantageous in arid systems, where soil‐borne microbial reservoirs are transient and spatially patchy (Placella et al. 2012; Maestre et al. 2015; Makhalanyane et al. 2015). By securing microbial continuity behaviorally, tortoises can stabilize digestion without the metabolic costs of physiological flexibility (Troyer 1982; Secor and Diamond 1998; Karasov and Martínez del Rio 2007). Such regulation is a likely driver of their persistence in landscapes defined by nutritional unpredictability and microbial scarcity (Henen 1998; Schwinning et al. 2004; Maestre et al. 2015). Coprophagy also serves a stoichiometric role, recovering residual organic matter, minerals, and microbial biomass that pass unabsorbed through the gut. In nutrient‐poor Tamaulipan thornscrub habitat (i.e., habitats characteristic of the geographical range of Texas tortoises), where forage is fibrous and nitrogen‐deficient, re‐ingestion of fecal material allows tortoises to extend the residence time of limiting nutrients within their bodies (Henen 1997; Bjorndal 1997). This behavioral stoichiometry parallels ecosystem models predicting tighter nutrient cycling under low fertility (Vitousek and Reiners 1975; Odum 1969; Sterner and Elser 2002). Through coprophagy, tortoises conserve energy and essential elements internally while reducing nutrient loss to decomposition (Hirakawa 2001; Bjorndal 1997; Sterner and Elser 2002).

Because Texas tortoises are long‐lived, the cumulative influence of such recycling behaviors can persist for decades (Judd and Rose 1983; Hellgren et al. 2000; Germano 1992). For example, over a tortoise's lifespan, repeated nutrient recapture may meaningfully contribute to the slow biogeochemical cycle that is characteristic of arid systems (Noy‐Meir 1973; Germano 1992). In this sense, Texas tortoises act not only as a consumer but as a regulator of elemental flow, linking organismal behavior to ecosystem‐scale processes. By selectively consuming feces from compatible sources, Texas tortoises behaviorally mediate both their internal and external microbial environments. Such selectivity reinforces beneficial microbial assemblages while minimizing exposure to incompatible or pathogenic taxa, thereby stabilizing digestive function through environmental variability. Additionally, the ingestion and redeposition of fecal material from multiple species may redistribute microbial communities across microhabitats, contributing to microbial connectivity and turnover in arid or semi‐arid ecosystems (Fierer and Jackson 2006; Maestre et al. 2015; Harris 2009). This coupling of behavior, microbial ecology, and nutrient transport demonstrates how individual decisions can influence emergent ecosystem function.

Recognizing coprophagy as an adaptive mechanism has practical implications for conservation and husbandry (Videvall et al. 2023; Kohl et al. 2014). In captivity or translocation programs, tortoises deprived of natural fecal exposure may experience microbial impoverishment and digestive inefficiency. Controlled exposure to conspecific feces or environmental microbial substrates could help maintain gut stability, analogous to probiotic management in other herbivorous reptiles. Conversely, attraction to omnivore feces such as raccoon scat underscores potential exposure to pathogens such as *Baylisascaris procyonis*, emphasizing the need for pathogen surveillance in management settings (Ogdee et al. 2017; Henke 2025).

The compatibility–risk gradient identified here likely shifts with season, diet, and community context. For example, during drought or periods of low plant quality, tortoises may strengthen preference for feces offering greater microbial or nutrient payoff. In areas where exotic omnivores like feral hogs are abundant, increased dietary overlap could further shape fecal selectivity, generating context‐dependent selection on cue use. Because coprophagy influences both internal symbioses and external nutrient fluxes, it may also create eco‐evolutionary feedbacks in which behavioral adaptations that enhance individual stability simultaneously reinforce nutrient retention and microbial exchange at the ecosystem level (Post and Palkovacs 2009; Jones et al. 1994). Testing these dynamics in natural settings would broaden the understanding of how behavioral and ecological processes co‐evolve in arid systems.

## 5 Conclusions

Coprophagy in the Texas tortoise is prevalent, selective, and evolutionarily meaningful. Captive individuals discriminate among feces types along a compatibility–risk gradient, integrating nutritional, microbial, and health cues to balance costs and benefits. This behavior enhances digestive efficiency, stabilizes symbiotic microbial communities, and promotes nutrient retention in resource‐limited environments. By behaviorally maintaining both internal symbiotic systems and external nutrient pathways, Texas tortoises demonstrate behavior that links physiology, ecology, and ecosystem function. Coprophagy thus represents an adaptive connection between organismal persistence and the slow, nutrient‐limited dynamics that define arid‐land ecosystems. However, selective coprophagy, as demonstrated in our study, was under controlled conditions, and until future research is conducted, extrapolations to wild tortoise behavior remain proposed hypotheses. Such future research could combine coprophagy with fecal chemistry and microbiome analysis to determine potential benefits from coprophagy.

## Author Contributions

**Christin Moeller:** conceptualization (equal), data curation (equal), investigation (equal), methodology (equal), writing – original draft (equal). **Scott E. Henke:** conceptualization (equal), data curation (equal), funding acquisition (equal), investigation (equal), methodology (equal), project administration (equal), resources (equal), supervision (equal), visualization (equal), writing – original draft (equal), writing – review and editing (equal). **Sandra Rideout‐Hanzak:** investigation (equal), methodology (equal), validation (equal), visualization (equal), writing – review and editing (equal). **Cord B. Eversole:** conceptualization (equal), data curation (equal), formal analysis (equal), funding acquisition (equal), investigation (equal), methodology (equal), validation (equal), visualization (equal), writing – original draft (equal), writing – review and editing (equal).

## Funding

This work was supported by NextDecade, NA. Rob and Bessie Welder Wildlife Foundation, NA.

## Ethics Statement

Research conducted within our study was conducted responsibly, lawfully, ethically, and safely. Study methods complied with all necessary ethical, legal, and conservation requirements as outlined by TAMUK IACUC protocol number 2021‐03‐08/1463.

## Conflicts of Interest

The authors declare no conflicts of interest.

## Supporting information


**Data S1:** ece373444‐sup‐0001‐Supplementaryfile.docx.

## Data Availability

All the required data are uploaded as Supporting Information—S1.
