# Supplementary material for: Behavioral Selection of Coprophagy in an Arid‐Adapted Herbivore: Does a Compatibility–Risk Gradient Shape Selective Coprophagy?
Source: Ecol Evol. 2026 Apr 7;16(4):e73444. doi: 10.1002/ece3.73444 (PMC13054235; doi:10.1002/ece3.73444)
Supplement: Supplementary file 1 — Data S1: ece373444‐sup‐0001‐Supplementaryfile.docx. [file ECE3-16-e73444-s001.docx]

**SUPPLEMENT**

File Code

set.seed(1)

pkgs <- c("readxl", "glmmTMB", "emmeans", "car", "DHARMa", "multcompView")

missing_pkgs <- pkgs[!vapply(pkgs, requireNamespace, logical(1), quietly = TRUE)]

if (length(missing_pkgs) > 0) {

stop("Missing required packages: ", paste(missing_pkgs, collapse = ", "))}

suppressPackageStartupMessages({

library(readxl)

library(glmmTMB)

library(emmeans)

library(car)

library(DHARMa)

library(multcompView)})

file_path <- "file_path_name"

outdir <- file.path(getwd(), "supp_outputs")

dir.create(outdir, showWarnings = FALSE, recursive = TRUE)

make_letters <- function(pair_df, level_names, alpha = 0.05) {

if (!("contrast" %in% names(pair_df))) stop("Missing 'contrast' column.")

if (!("p.value" %in% names(pair_df))) stop("Missing 'p.value' column.")

pair_df$contrast <- as.character(pair_df$contrast)

pm <- matrix(1, nrow = length(level_names), ncol = length(level_names),

dimnames = list(level_names, level_names))

for (i in seq_len(nrow(pair_df))) {

ab <- strsplit(pair_df$contrast[i], " - ", fixed = TRUE)[[1]]

if (length(ab) != 2) next

a <- ab[1]; b <- ab[2]

if (!(a %in% level_names) || !(b %in% level_names)) next

p <- suppressWarnings(as.numeric(pair_df$p.value[i]))

if (!is.finite(p)) p <- 1

pm[a, b] <- p; pm[b, a] <- p}

nosig <- pm > alpha

let <- multcompView::multcompLetters(nosig)$Letters

data.frame(ScatType = level_names, .group = let[level_names], row.names = NULL)}

wide <- read_excel(file_path)

scat_cols <- c("Self tortoise", "Other tortoise", "Raccoon","Coyote", "Feral hog", "Nilgai")

st <- stack(wide[scat_cols])

names(st) <- c("Area", "ScatType")

long <- data.frame(

TortoiseID = factor(rep(wide[["Tortoise number"]], times = length(scat_cols))),

Sex = factor(rep(wide[["Sex"]], times = length(scat_cols))),

ScatType = factor(st$ScatType,

levels = c("Self tortoise", "Other tortoise",

"Feral hog", "Raccoon", "Coyote", "Nilgai")),

Area = as.numeric(st$Area))

long$Eaten <- as.integer(long$Area > 0)

sink(file.path(outdir, "Data_summary.txt"))

print(table(long$ScatType, long$Eaten))

print(aggregate(Area ~ ScatType, long, mean))

sink()

m1_full <- glmmTMB(

Eaten ~ ScatType * Sex + (1 | TortoiseID),

family = binomial,

data = long)

a1_full <- car::Anova(m1_full, type = "III")

use_simple_m1 <- FALSE

if ("ScatType:Sex" %in% rownames(a1_full)) {

p_int <- a1_full["ScatType:Sex", "Pr(>Chisq)"]

if (is.finite(p_int) && p_int > 0.1) use_simple_m1 <- TRUE}

m1 <- if (use_simple_m1) {glmmTMB(

Eaten ~ ScatType + Sex + (1 | TortoiseID),

family = binomial,

data = long )} else m1_full

a1 <- car::Anova(m1, type = "III")

sink(file.path(outdir, "Model1_summary.txt"))

print(summary(m1))

print(a1)

sink()

emm1 <- emmeans(m1, ~ ScatType, type = "response")

pair1s <- as.data.frame(summary(pairs(emm1, adjust = "tukey")))

levs1 <- as.character(as.data.frame(emm1)$ScatType)

letters1 <- make_letters(pair1s, levs1)

cld1 <- merge(as.data.frame(emm1), letters1, by = "ScatType", all.x = TRUE)

write.csv(as.data.frame(emm1), file.path(outdir, "Table_emm_probability.csv"), row.names = FALSE)

write.csv(pair1s, file.path(outdir, "Table_pairs_probability.csv"), row.names = FALSE)

write.csv(cld1, file.path(outdir, "Table_cld_probability.csv"), row.names = FALSE)

sim1 <- DHARMa::simulateResiduals(m1, n = 1000)

png(file.path(outdir, "Fig_M1_DHARMa_residuals.png"), width = 1100, height = 800, res =150)

plot(sim1)

dev.off()

capture.output (list(testDispersion(sim1), testZeroInflation(sim1), testOutliers(sim1)),

file = file.path(outdir, "Diag_M1_DHARMa_tests.txt"))

pos <- long[long$Area > 0, , drop = FALSE]

m2_full <- glmmTMB(Area ~ ScatType * Sex + (1 | TortoiseID),family = Gamma(link = "log"), data = pos)

a2_full <- car::Anova(m2_full, type = "III")

use_simple_m2 <- FALSE

if ("ScatType:Sex" %in% rownames(a2_full))

{p_int2 <- a2_full["ScatType:Sex", "Pr(>Chisq)"]

if (is.finite(p_int2) && p_int2 > 0.1) use_simple_m2 <- TRUE}

m2 <- if (use_simple_m2) {glmmTMB(Area ~ ScatType + Sex + (1 | TortoiseID), family = Gamma(link = "log"), data = pos)} else m2_full

a2 <- car::Anova(m2, type = "III")

sink(file.path(outdir, "Model2_summary.txt"))

print(summary(m2))

print(a2)

sink()

emm2 <- emmeans(m2, ~ ScatType, type = "response")

pair2s <- as.data.frame(summary(pairs(emm2, adjust = "tukey")))

levs2 <- as.character(as.data.frame(emm2)$ScatType)

letters2 <- make_letters(pair2s, levs2)

cld2 <- merge(as.data.frame(emm2), letters2, by = "ScatType", all.x = TRUE)

write.csv(as.data.frame(emm2), file.path(outdir, "Table_emm_amount.csv"), row.names = FALSE)

write.csv(pair2s, file.path(outdir, "Table_pairs_amount.csv"), row.names = FALSE)

write.csv(cld2, file.path(outdir, "Table_cld_amount.csv"), row.names = FALSE)

sim2 <- DHARMa::simulateResiduals(m2, n = 1000)

png(file.path(outdir, "Fig_M2_DHARMa_residuals.png"), width = 1100, height = 800, res =150)

plot(sim2)

dev.off()

capture.output(

list(testDispersion(sim2), testOutliers(sim2)),

file = file.path(outdir, "Diag_M2_DHARMa_tests.txt"))

sink(file.path(outdir, "SessionInfo.txt"))

sessionInfo()

sink()
